# Graph attention spatio-temporal graph neural network based human–light interaction modeling for indoor lighting environments

**DOI:** 10.1371/journal.pone.0358774

**Published:** 2026-09-25

**Authors:** Ting Gao, Lingling Sun

**Affiliations:** Academy of Art and Media Design, NanChang Institute of Science and Technology, NanChang, Jiangxi, China; University of Essex Faculty of Science and Engineering, UNITED KINGDOM OF GREAT BRITAIN AND NORTHERN IRELAND

## Abstract

In indoor light environment design, the dynamic interaction between human behavior and lighting systems has an important impact on environmental perception results. However, existing methods are difficult to describe its spatial structure and temporal evolution characteristics at the same time. To this end, this paper constructs a human-light spatio-temporal relationship modeling method based on graph attention spatio-temporal graph neural network, which uniformly represents human body nodes and lamp nodes as spatio-temporal graph structures, and introduces heterogeneous graph attention and adaptive temporal attention mechanisms for joint modeling. The study conducted experimental verification on real indoor scene data for 7 consecutive days and a total of 302,400 time steps. The results show that the proposed model achieves MAE of 0.096 ± 0.010, MSE of 0.017 ± 0.004, RMSE of 0.130 ± 0.015, and R² of 0.943 ± 0.018 in the environment perception prediction task, which is significantly better than various comparison models. At the same time, the environmental perception results show stable and continuous evolution characteristics in the space and time dimensions. Research shows that modeling of human-light spatiotemporal relationships based on graph attention can effectively improve the accuracy and interpretability of indoor light environment perception, and provides important methodological support for intelligent light environment design and human factor-driven control.

## 1. Introduction

With the development of smart buildings and human-centered design, dynamic control of indoor light environment has become the key to balancing comfort and energy efficiency. Lighting not only affects visual perception, but also regulates human body rhythm and cognitive status [6]. Advances in photoelectric sensing and artificial intelligence promote the development of lighting systems from static to adaptive, human-centered intelligent modes [5]. However, existing strategies mostly focus on lighting or energy consumption optimization and lack systematic modeling of human-light interaction, resulting in lagging light environment response and insufficient adaptability [16]. To this end, this study constructs a human-light spatiotemporal interaction modeling framework to reveal the dynamic coupling mechanism of behavior and lighting response to support intelligent lighting design driven by environmental perception.

In recent years, research on indoor light environment modeling and intelligent sensing has made significant progress. The proposal of high dynamic range illumination estimation and spatiotemporal consistency prediction methods has improved the accuracy and continuity of indoor light field reconstruction, providing support for virtual and augmented reality applications [12]. Research also shows that there are significant differences in the perception of indoor environments by different groups of people, promoting the concept of personalized lighting design with user perception as the core [22]. In terms of modeling methods, graph neural network (GNN) shows high efficiency in processing spatiotemporal dependencies, providing a new way to express multi-agent interactive relationships in complex indoor environments [11]. The introduction of the spatiotemporal graph convolution structure further enhances the model’s ability to identify and classify the collaborative behaviors of heterogeneous subjects [7]. In addition, the behavior recognition accuracy of deep learning under low illumination conditions has been significantly improved, laying the foundation for perception modeling in complex light environments [25]. Although research has made progress in light environment modeling and perception optimization, there is still a lack of deep modeling of the systematic spatiotemporal expression and heterogeneous relationships of human-light interactions.

The main contributions of this study are summarized as follows:

- A spatio-temporal relationship modeling method for human-light interaction in indoor lighting environments is proposed, which overcomes the limitation of traditional lighting environment studies that mainly focus on single environmental parameters or static lighting state analysis, and achieves unified representation of human behavioral changes and luminaire response processes.
- A graph attention-based spatio-temporal modeling framework integrating heterogeneous relationship modeling and dynamic temporal dependency analysis is constructed, which enhances the capability of the model to characterize interaction differences among different entities and the dynamic evolution patterns of lighting environments.
- The effectiveness of the proposed method is validated using continuously collected human-light interaction data from real indoor scenarios, demonstrating the potential of spatio-temporal relationship modeling in improving environmental perception accuracy and enhancing model interpretability.

This study constructs a modeling framework based on graph attention spatiotemporal graph neural network (GAT-STGNN), and achieves dynamic representation and key relationship identification of human-light interaction by introducing heterogeneous attention and adaptive time modeling mechanisms. The research results verify the accuracy and stability of the model in environmental perception prediction, and provide new technical paths and application value for intelligent lighting design. The research results help promote the transformation of indoor light environment from passive control to active perception, and provide theoretical support and practical reference for realizing intelligent lighting design that pays equal attention to high comfort and high energy efficiency.

## 2. Literature review

With the integration of smart buildings and human-centered design concepts, indoor light environment research is developing from visual lighting to perception-driven and behavioral response [2, 4]. Lighting design gradually goes beyond static illumination control and begins to integrate multi-dimensional factors such as spatial structure, psychological feelings and activity characteristics. In recent years, research has promoted the transformation of light environment from quantitative measurement to intelligent perception modeling by integrating physical lighting parameters and human perception mechanisms. This trend has formed a research framework with light physical characteristics, human behavior and spatiotemporal neural modeling as the core.

Early studies on lighting environments mainly focused on physical parameters such as illuminance, luminance, and spatial configuration, with emphasis on analyzing the relationship between lighting environment characteristics and visual perception. Miki et al. [15] conducted an evaluation study on lighting impressions in residential spaces by analyzing the relationships between light distribution characteristics and spatial perception, comfort, and preference, and explored the influence of different light distribution patterns on users’ spatial experiences. This study was primarily based on subjective evaluation data under static lighting conditions and focused on the association between the spatial distribution characteristics of lighting environments and perceptual outcomes. Kim et al. [10] developed a comprehensive indoor lighting environment evaluation framework from the perspectives of user comfort and health requirements, and proposed an evaluation procedure involving environmental assessment, perception analysis, and result comparison to support the analysis of lighting design effects. Ruan et al. [17] combined image brightness feature analysis with physical modeling methods to establish a predictive relationship between image-based indicators and perceived indoor spatial luminance, providing a data-driven approach for lighting environment evaluation based on visual information. This study mainly relied on image features for luminance perception prediction and did not further consider the influence of changes in human spatial positions on lighting environment states. Achsani et al. [1] analyzed the effects of building spatial layout, regional location, viewing direction, and temporal variations on visual comfort through field measurements, and investigated the relationship between spatial conditions and daylight environment experiences. The above studies have enriched indoor lighting environment evaluation methods from the perspectives of physical parameters, subjective perception, and spatial factors. However, they mainly focused on lighting environment states or visual experience analysis, while the dynamic interaction relationship between human behavioral changes and lighting responses still requires further investigation.

Based on lighting environment evaluation studies, some researchers have further incorporated human perception and behavioral factors into indoor and outdoor environmental analysis, focusing on the relationship between spatial environment characteristics and user experiences. Chen et al. [3] analyzed visual perception in nighttime street environments based on computer vision and perceptual evaluation methods, and explored the relationship between environmental characteristics and subjective evaluations such as perceived safety and comfort, providing an analytical approach for nighttime environment perception evaluation. Wei et al. [21] investigated the effects of indoor and outdoor fitness environmental factors on residents’ activity intensity through questionnaire surveys and structural equation modeling, and analyzed the associations between spatial environment, safety, convenience, comfort, and behavioral performance. Zhao [24] investigated the influence of body movement processes on spatial perception from the perspective of embodied cognition by combining experimental data analysis, and explored the interaction between human activities and environmental experiences. Sui [18] developed an indoor environment visualization and interaction analysis platform based on Web-Based Three-Dimensional Technology (Web3D) technology, enabling visual associations among spatial schemes, environmental parameters, and user perception evaluations. The above studies indicate that human behavior and subjective perception have gradually become important factors in environmental evaluation. However, related studies have mainly focused on environmental experience analysis or interactive visualization, while unified modeling of human behavioral states, lighting system responses, and their spatio-temporal relationships remains limited.

With the increasing dimensions of environmental perception data, some studies have further introduced graph-based structures and spatio-temporal modeling methods to describe multi-entity correlation relationships in complex systems. Graph Neural Networks (GNNs) and their extended models can represent relationships among entities through node and edge structures, providing new methodological approaches for modeling complex interaction processes. Liu and Han [13] developed an intelligent office lighting system based on personnel detection, environmental sensing, and communication control, in which personnel location information was obtained through object detection methods and dynamic lighting adjustment was achieved by integrating a lighting control module. Ji et al. [9] proposed an indoor light and thermal estimation method based on visible-light panoramic images, which predicted indoor light and thermal distribution states by combining image information with physical models. Tew et al. [20] proposed the Deep Spatio-Temporal Hypergraph Convolutional Neural Network for Soft Sensing (ST-HCSS) spatio-temporal hypergraph convolutional network, which describes high-order correlation relationships in multivariate systems through hypergraph structures for measurement tasks. Mateos-Aparicio-Ruiz et al. [14] developed a human-machine collaborative decision-making model based on spatio-temporal graph neural networks to analyze relationship variations during dynamic decision-making processes. The above studies demonstrate that graph-based structures and spatio-temporal modeling methods have been applied in multi-entity relationship analysis and dynamic system prediction. However, existing methods mainly focus on general relationship modeling or specific application tasks, while the joint modeling of heterogeneous relationships between humans and luminaires and their spatio-temporal evolution processes in indoor environments still requires further investigation.

To further summarize the development of existing studies in indoor lighting environment evaluation, human behavior perception, and spatio-temporal relationship modeling, this study reviews and categorizes relevant literature from three aspects: research methods, research focuses, and major research boundaries, as presented in Table 1.

**Table 1:** Comparison of existing studies on indoor lighting environment and related spatio-temporal modeling.

Reference	Methodology	Research focus	Limitation
Miki et al. [15]	Subjective evaluation experiments and structural equation modeling	Analyzing the influence of light distribution on spatial perception and lighting preference	Mainly focuses on static lighting conditions without considering behavioral dynamics
Kim et al. [10]	Literature analysis and comprehensive evaluation framework	Developing an evaluation method for lighting environment, occupant comfort, and well-being	Lacks long-term measurements and dynamic interaction modeling
Ruan et al. [17]	Image feature extraction and brightness prediction model	Establishing the mapping between image metrics and perceived spatial brightness	Image-based approach does not consider occupant position changes
Achsani et al. [1]	Field measurements and visual comfort analysis	Investigating the effects of spatial layout and temporal variations on visual comfort	Limited experimental scenarios without artificial lighting control
Chen et al. [3]	Computer vision and perception evaluation model	Evaluating visual perception characteristics of nighttime environments	Focuses on outdoor environments, which differs from indoor lighting conditions
Wei et al. [21]	Questionnaire survey and structural equation modeling	Analyzing the influence of environmental factors on activity behavior	Relies on subjective evaluations with limited dynamic environmental data
Zhao [24]	Virtual-real integrated experiments and behavioral perception analysis	Exploring the influence of body movement on spatial perception	Does not establish the relationship between human behavior and lighting response
Sui [18]	Web3D visualization and interactive evaluation	Developing visualization-based analysis for indoor environment design	Based on virtual environments with limited real-world operational data
Liu & Han [13]	Object detection and intelligent lighting control system	Implementing personnel-aware lighting control based on occupant detection	Focuses on control implementation rather than spatio-temporal relationship modeling
Ji et al. [9]	Panorama-based image analysis and light–thermal estimation model	Estimating indoor light and thermal distributions from visual information	Does not consider occupant behavior and dynamic lighting effects
Tew et al. [20]	Spatio-temporal hypergraph convolutional neural network	Modeling high-order spatio-temporal relationships in multivariate systems	Developed for industrial processes rather than indoor environments
Mateos-Aparicio-Ruiz et al. [14]	Spatio-temporal graph neural network and meta-learning	Modeling dynamic relationships in human–AI collaborative decision-making	Applied to decision tasks without physical environment interaction

As shown in Table 1, existing studies have conducted systematic investigations from the perspectives of lighting environment parameter analysis, visual perception evaluation, and intelligent lighting control, and have gradually introduced spatio-temporal modeling methods such as graph neural networks. However, most existing studies focus on single environmental factors, perception evaluation, or control tasks, and the unified representation of dynamic interaction relationships between human behaviors and luminaire states still requires further investigation. Therefore, this study develops a human-light relationship modeling method based on a graph attention spatio-temporal graph neural network, which achieves dynamic modeling of indoor lighting environment perception processes by integrating spatial correlations, temporal evolution, and heterogeneous interaction features.

## 3. Research design

### 3.1 Data sources and research scenarios

This study focuses on the spatiotemporal modeling of human-light interaction relationships in indoor light environment design, and constructs a data collection and modeling experimental environment for typical indoor usage scenarios. To ensure the authenticity of experimental data and the reliability of model validation, this study obtained human-light interaction data through actual indoor environment monitoring. The experimental system mainly consists of a human position perception module, a lighting status acquisition module, and a data synchronization acquisition unit. Among them, human node information was collected using a Ultra-Wideband (UWB) indoor positioning system (DWM1001-DEV, Qorvo Inc., USA). Real-time spatial coordinate information of occupants was obtained by deploying positioning anchors and tags. The positioning accuracy of this system can reach within 10 cm, which satisfies the requirements for monitoring indoor occupant movement trajectories. Luminaire node information was obtained through an intelligent lighting acquisition module. An illuminance sensor (BH1750FVI, ROHM Semiconductor, Japan) was used to collect changes in environmental illuminance, while the on/off status and operating parameters of luminaires were recorded through an intelligent lighting control module. The measurement range of the illuminance sensor is 1–65535 lx, with a sampling accuracy of 1 lx. All sensor data were synchronously collected through an STM32F407 data acquisition terminal, and data alignment was performed based on unified timestamps to ensure temporal consistency between human behavioral changes and luminaire response processes.

Since the indoor lighting environment state is mainly characterized by spatial illuminance levels, this study selected the real-time horizontal illuminance value (illuminance, lx) in the human activity area as the environmental perception target variable to describe the dynamic variation process of the indoor lighting environment under human-light interactions. The illuminance data were directly measured using the BH1750FVI illuminance sensor. The sensor was installed in the main occupant activity area to continuously record illuminance variations under different temporal states with a sampling interval of 1 s. After time synchronization processing, these data were used as target observation data for model training and validation.

The experimental data acquisition system was deployed in a real indoor environment, with an experimental area of approximately 8 m × 6 m × 3 m. The environment contained 6 human nodes and 8 luminaire nodes. A high-performance computing workstation was used as the data storage and model training platform, with hardware configurations including an Intel Core i9-13900K processor, an NVIDIA RTX 4090 GPU (24 GB memory), and 64 GB Fifth-Generation Double Data Rate (DDR5) Random-Access Memory (RAM). The software environment was developed based on Python 3.10, with PyTorch 2.1.0 adopted as the deep learning framework. In addition, NumPy 1.26.0, Pandas 2.1.0, and Scikit-learn 1.3.0 were integrated for data processing, model training, and performance evaluation.

Before data collection, all participants were informed of the research objectives, experimental procedures, and data usage methods, and signed informed consent forms prior to participation. Data collection is carried out within a continuous period of time, covering the complete process of personnel activities and lighting status changes. The experimental data collection duration is 7 days, and the daily collection time is 08:00–20:00. The time sampling interval is set to 1s to fully characterize the timing characteristics of indoor personnel behavior changes and lamp status adjustments. Ultimately, continuous and uninterrupted human-light interaction time series data is formed, providing a stable data basis for spatiotemporal modeling. In terms of data composition, the dataset mainly consists of two types of data: human behavior data and luminaire operating state data. Specifically, at each time step t, a single sample is composed of the states of 14 nodes, including 6 human nodes and 8 luminaire nodes. Each human node contains three-dimensional spatial coordinates (x, y, z) and human activity state features, while each luminaire node contains luminaire switching states, illuminance response parameters, and operating state information. Therefore, the sample at a single time step can be represented as a state set jointly composed of a human node feature matrix and a luminaire node feature matrix, which is used to describe the current indoor human-light interaction state.

After completing the original data acquisition, unified data preprocessing operations were performed, including time synchronization, anomaly detection, missing value correction, and data normalization. First, timestamp calibration was performed on data collected from different sensors, and the human behavior data and luminaire operating data were matched one-to-one through a unified sampling interval. Second, potential anomalies during sensor acquisition were processed, mainly including sudden changes caused by temporary sensor failures, data points exceeding reasonable physical ranges, and duplicate records generated by communication anomalies. Anomaly determination was mainly based on sensor measurement ranges and continuous temporal variation constraints. When data exceeded the valid measurement range of the device, or the variation magnitude between adjacent time steps exceeded the predefined threshold, the sample was identified as abnormal and corrected using interpolation within adjacent time windows. For missing data, linear interpolation was applied when the continuous missing duration was less than 5 sampling periods. When the continuous missing duration exceeded 5 sampling periods, the corresponding time segments were removed to avoid excessive compensation affecting model training performance. After anomaly processing and missing value correction, input features were normalized to map data with different dimensions into a unified range, thereby improving the stability of model training. After processing, the final constructed data set contains a total of approximately 302,400 time step samples, each sample corresponding to a complete human-light interaction state, which is used for subsequent spatiotemporal relationship modeling and model training. In order to ensure the objectivity and generalization ability of model evaluation, the data set is divided into training set, verification set and test set in chronological order, with the proportions of 70%/15%/15% respectively.

Considering the strong autocorrelation of the continuously sampled data at 1-s intervals over short time scales, this study adopted a chronological contiguous-block partitioning strategy rather than randomly shuffling the samples before data partitioning. The training, validation, and test sets corresponded to mutually independent contiguous time segments, and the model input sequences were constructed only within their respective data subsets, without allowing temporal windows to cross the partition boundaries between different subsets. This strategy prevents the same temporal window or directly overlapping temporal information from being simultaneously included in the training and test sets, thereby reducing the risk of information leakage caused by highly correlated samples under continuous sampling conditions. The test set was always positioned chronologically after the training and validation data; therefore, the reported test performance reflects the model’s predictive capability for subsequent time periods that were not involved in model training.

**Fig 1:**
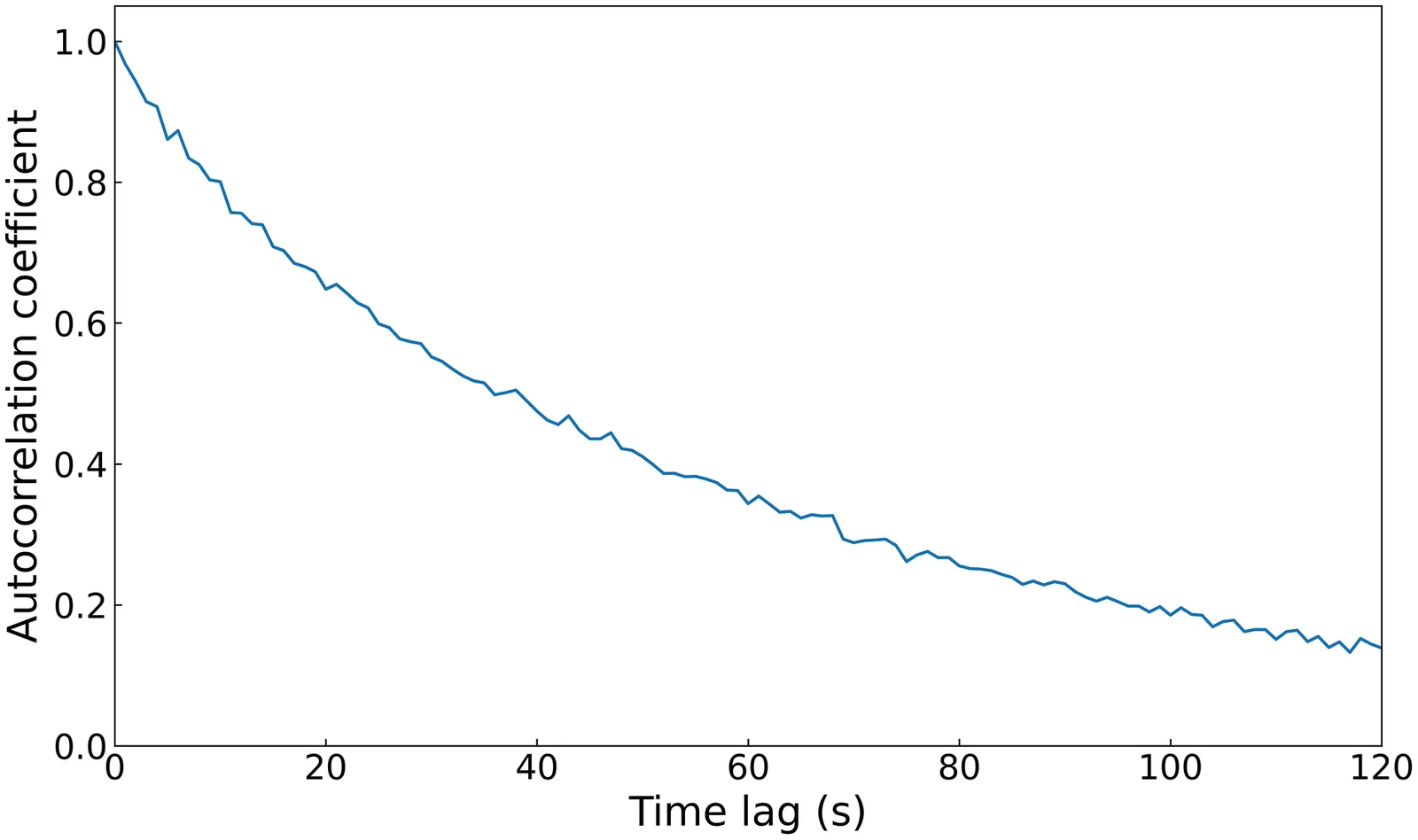
Autocorrelation analysis of indoor illuminance time series.

Since the data collected in this study exhibits continuous time-series characteristics, an autocorrelation analysis method was further adopted to evaluate the temporal dependency of the environmental perception target variable. The autocorrelation function is used to describe the correlation degree between data at the current time step and states with different time lags, and its calculation results can reflect the temporal continuity of lighting environment states. In this study, the autocorrelation coefficients of the illuminance sequence within a time lag range of 0–120 s were calculated, and the results are shown in Fig 1.

As shown in Fig 1, the indoor illuminance sequence exhibits significant temporal correlation at short time scales. The autocorrelation coefficient remains at a high level under small time lags and gradually decreases with increasing time lag. This indicates that indoor lighting environment variations exhibit continuous evolution characteristics, which are consistent with the dynamic variation patterns of occupant movement, luminaire state adjustments, and environmental response processes. Meanwhile, as the time interval increases, the correlation between illuminance states gradually weakens, indicating that environmental states are not completely determined by short-term historical information. Therefore, adopting a spatio-temporal graph modeling method that simultaneously considers temporal features and human-light spatial interaction relationships is reasonable.

This short-term correlation mainly reflects the dynamic evolution characteristics of the continuous indoor lighting environment. To avoid its potential influence on model performance evaluation, this study did not adopt random sample partitioning. Instead, mutually non-overlapping contiguous data subsets were constructed in chronological order, and model input windows were restricted from crossing dataset boundaries, thereby preventing the training process from directly utilizing information from the test time period.

### 3.2 Human-light spatio-temporal relationship modeling

In order to describe the spatial structure and time evolution characteristics of the human-light interaction relationship in indoor light environment design, this paper abstracts the indoor environment into a spatio-temporal graph structure composed of human body nodes and lamp nodes. This modeling method can express the interactive relationships between different subjects under a unified framework, and provide structured input for subsequent representation learning based on graph neural networks.

The indoor environment operates at discrete time steps t∈{1,2,…,T}, where T represents the total number of time steps. Define the human body node set and the lamp node set as follows:


$$\begin{array}{c} V_{h}=\{v_{\text{1}}^{h},v_{\text{2}}^{h},\ldots ,v_{N_{h}}^{h}\},V_{l}=\{v_{\text{1}}^{l},v_{\text{2}}^{l},\ldots ,v_{N_{l}}^{l}\} \end{array}$$ (1)

Among them, N_h_ represents the number of human body nodes, and N_l_ represents the number of lamp nodes. Combine two types of nodes to build a unified node collection:


$$\begin{array}{c} V=V_{h}\cup V_{l} \end{array}$$ (2)

At each time step t, node v_i_∈V corresponds to a feature vector $x_{i}^{\left(t\right)}$, which is used to describe the status information of the node at the current moment. The node characteristics of the human body mainly reflect its spatial position and behavioral status, while the node characteristics of the lamps are used to describe its operating status and light environment-related parameters.

In order to describe the spatial relationship between the human body and lamps, this paper constructs a spatial relationship graph based on the node set V. Define the spatial relationship edge set as:


$$\begin{array}{c} E_{s}=\{(v_{i},v_{j})|v_{i}\in V_{h},\text{\textbackslash{}hspace\{0.33em}\}v_{j}\in V_{l}\} \end{array}$$ (3)

Each edge represents the potential interaction relationship between human body nodes and lamp nodes in the spatial dimension. Based on spatial position constraints, the adjacency matrix $A_{s}\in R^{\left|V\right|\times \left|V\right|}$ is introduced, and its elements are defined as:


$$\begin{array}{c} A_{ij}^{s}=\left\{ \begin{array}{cc} @ll@1, & \text{if }d_{ij}\leq \delta \\ 0, & \text{otherwise} \end{array}\right. \end{array}$$ (4)

Among them, d_ij_ represents the Euclidean distance between the human body node v_i_^h^ and the lamp node v_j_^l^, and δ is the distance threshold, which is used to limit the effective human-light spatial interaction range. In this way, the spatial correlation structure with practical significance can be preserved while ensuring computational efficiency.

Considering that human body behavior and lighting status dynamically change over time, it is difficult to completely describe the human-light interaction process by relying only on static spatial images. Therefore, this paper further introduces the time dimension and expands the spatial relationship graph into a spatio-temporal graph structure.

In the time dimension, a self-connecting edge across time steps is established for each node v_i_ to describe the continuous evolution relationship of the node state. Define the time relationship edge set as:


$$\begin{array}{c} E_{t}=\{(v_{i}^{\left(t\right)},v_{i}^{\left(t+1\right)})|v_{i}\in V,\text{\textbackslash{}hspace\{0.33em}\}t=1,\ldots ,T-1\} \end{array}$$ (5)

On this basis, the construction of the space-time graph G is expressed as:


$$\begin{array}{c} G\text{= (}V\text{,}E_{s}\text{,}E_{t}\text{)} \end{array}$$ (6)

This graph structure can simultaneously express the spatial interaction relationship between the human body and the lamp, as well as the dynamic evolution characteristics of each node state over time.

On the space-time graph G, the state of each node at time step t is represented by the feature vector $x_{i}^{\left(t\right)}$. In order to facilitate subsequent model processing, the node features are stacked along the time dimension to form a time series feature matrix:


$$\begin{array}{c} X_{i}=[x_{i}^{\left(1\right)},x_{i}^{\left(2\right)},\ldots ,x_{i}^{\left(T\right)}] \end{array}$$ (7)

Further, the feature representations of all nodes are summarized into the overall input tensor:


$$\begin{array}{c} X=\{X_{i}|v_{i}\in V\} \end{array}$$ (8)

This input form provides a unified data interface for subsequent graph attention spatiotemporal graph neural networks, enabling the model to learn the temporal representation of human-light interaction relationships under spatial structure constraints.

### 3.3 Graph attention spatiotemporal graph neural network

**Fig 2:**
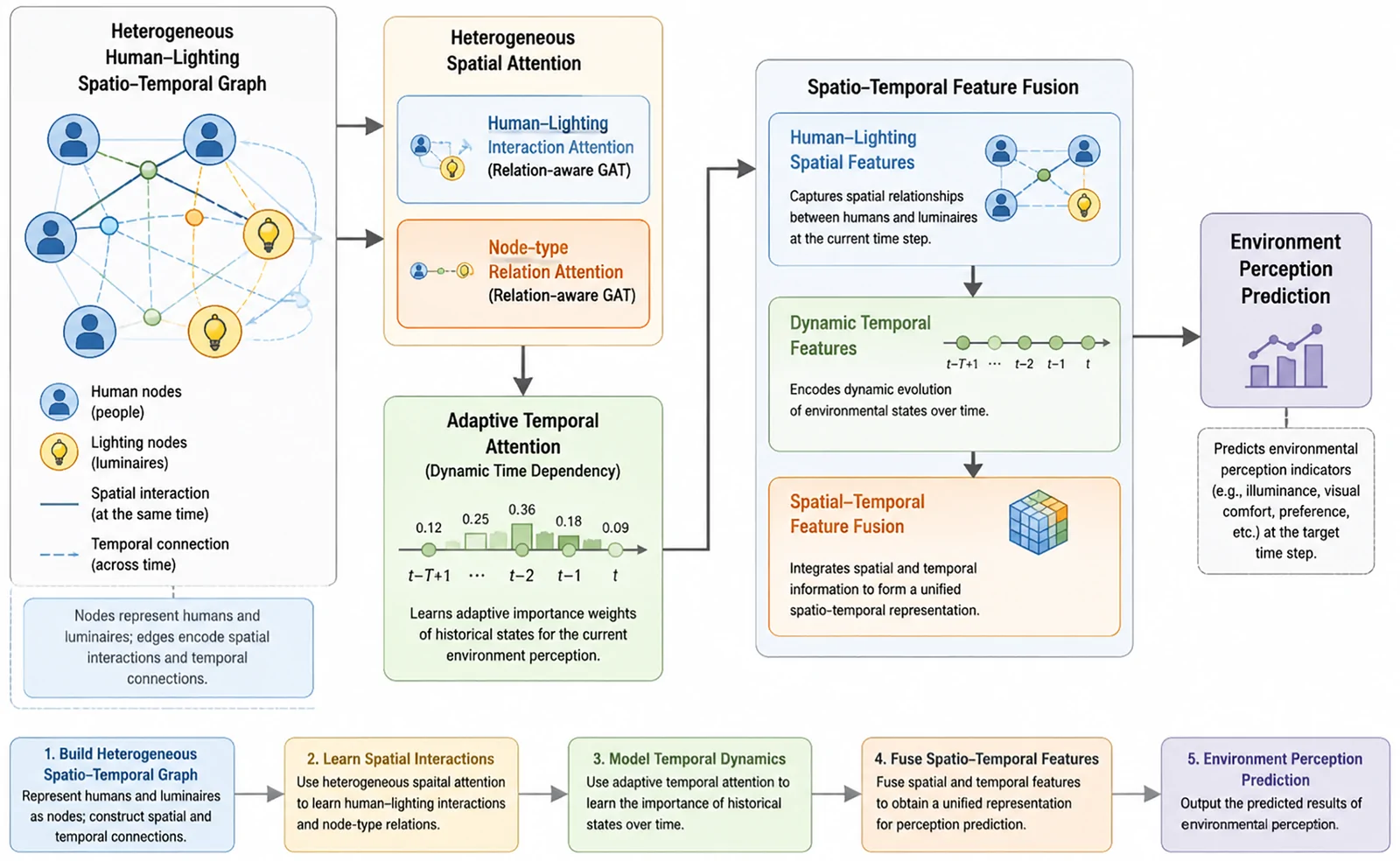
GAT-STGNN model structure diagram.

After completing the formal modeling of the human-light spatiotemporal relationship, this paper further constructed a spatio-temporal graph neural network (GraphAttention–basedSpatio-TemporalGraphNeuralNetwork, GAT-STGNN) based on the graph attention mechanism, which is used to learn the environment-aware representation under the complex human-light interaction relationship. Different from the standard spatiotemporal graph neural network, this model does not directly follow the existing graph attention or temporal modeling framework. Instead, it makes targeted improvements in the attention calculation mechanism and spatiotemporal modeling method in view of the characteristics of strong spatial heterogeneity, significant differences in subject types, and uneven temporal impact in human-light interaction. The specific model structure diagram is shown in Fig 2.

In standard graph attention networks, the influence of adjacent nodes on the target node is usually modeled by a unified form of attention function, which defaults to homogeneity of all nodes at the semantic level. However, in human-light interaction scenarios, there are significant functional and semantic differences between human body nodes and lamp nodes. If a unified attention calculation method is adopted, it is easy to weaken the expressive ability of the key human-light relationship. To this end, this paper introduces a heterogeneous graph attention mechanism based on node type distinction. The input feature of node vi at time step t is $x_{i}^{\left(t\right)}$, which is first projected to the latent space through linear mapping:


$$\begin{array}{c} h_{i}^{\left(t\right)}=Wx_{i}^{\left(t\right)} \end{array}$$ (9)

where W is the learnable weight matrix. Subsequently, the attention weight between node v_i_ and its neighbor node v_j_ is calculated. Different from the unified attention function in standard GAT, this paper introduces a relationship-aware term according to the node type (human-light), and defines the attention coefficient as:


$$\begin{array}{c} e_{ij}^{\left(t\right)}=a^{T}\left[h_{i}^{\left(t\right)}\;\|\;h_{j}^{\left(t\right)}\;\|\;r_{ij}\right] \end{array}$$ (10)

where a is a learnable parameter vector, and r_ij_ is a coding item representing the type of human-light relationship, which is used to explicitly distinguish the interactive relationships between different subjects. Normalize the attention coefficients of all neighbor nodes to obtain the final spatial attention weight:


$$\begin{array}{c} \alpha_{ij}^{\left(t\right)}=\frac{exp\left(e_{ij}^{\left(t\right)}\right)}{\sum_{k\in N\left(i\right)}exp\left(e_{ik}^{\left(t\right)}\right)} \end{array}$$ (11)

This improvement enables the model to explicitly perceive human-light heterogeneous relationships during the attention computation stage, rather than relying solely on feature similarity for implicit differentiation.

After obtaining the spatial attention weight, this paper performs weighted aggregation on node neighborhood features to form a spatially aware representation. The spatial aggregation feature of node v_i_ at time step t is expressed as:


$$\begin{array}{c} z_{i}^{\left(t\right)}=\sum_{j\in N\left(i\right)}\alpha_{ij}^{\left(t\right)}h_{j}^{\left(t\right)} \end{array}$$ (12)

Compared with standard graph attention networks, this aggregation process incorporates human-light relationship information into the attention weights, making the aggregation results more focused on interactive relationships that contribute more to environmental perception.

In terms of time series modeling, standard spatiotemporal graph neural networks usually use fixed time windows or recurrent neural network structures to model node states. By default, different time steps have the same impact on the current state. However, in indoor light environments, the influence of human behavior and lighting status has obvious time attenuation characteristics, and the contribution of different historical moments to the current perception results is not consistent. To this end, this paper introduces an adaptive temporal attention mechanism to perform weighted modeling of the historical states of nodes at different time steps. The spatial feature of node v_i_ within the time window τ∈{t − K + 1,…,t} is $z_{i}^{\left(\tau \right)}$, and its temporal attention weight is defined as:


$$\begin{array}{c} \beta_{i}^{\left(\tau \right)}=\frac{exp\left(q^{T}z_{i}^{\left(\tau \right)}\right)}{\sum_{s=t-K+1}^{t}exp\left(q^{T}z_{i}^{\left(s\right)}\right)} \end{array}$$ (13)

where q is the learnable temporal attention parameter vector. Based on this weight, the timing of the node at time step t is expressed as:


$$\begin{array}{c} s_{i}^{\left(t\right)}=\sum_{\tau =t-K+1}^{t}\beta_{i}^{\left(\tau \right)}z_{i}^{\left(\tau \right)} \end{array}$$ (14)

This mechanism can adaptively adjust the influence weights of different time steps according to the importance of historical states, avoiding information redundancy or noise interference caused by fixed-time modeling.

After completing the spatial and temporal feature modeling, this paper uses the temporal representation of nodes as the final human-light spatio-temporal fusion feature for subsequent environment perception tasks. Summarizing the output representation of all nodes at time step t can be expressed as:


$$\begin{array}{c} S^{\left(t\right)}=\{s_{i}^{\left(t\right)}|v_{i}\in V\} \end{array}$$ (15)

This representation simultaneously encodes human-light spatial structure information, heterogeneous interaction relationships, and temporal evolution characteristics, providing high-quality input for subsequent environmental perception predictions.

After completing the modeling of the spatial attention and temporal attention mechanisms, this article uses an end-to-end joint optimization strategy to uniformly train the model parameters. All learnable parameters in the model, including spatial attention weights, temporal attention weights, and feature mapping matrices, are updated through the same optimization goal, thereby ensuring the overall consistency of the spatiotemporal feature learning process. For the environment-aware prediction task, this paper uses the mean square error as the loss function of model training to measure the difference between the model prediction results and the real observation values. The loss function is defined as:


$$\begin{array}{c} L=\frac{\text{1}}{T}\sum_{t=1}^{T}{\|{\hat{y}}^{\left(t\right)}-y^{\left(t\right)}\|}_{\text{2}}^{\text{2}} \end{array}$$ (16)

Among them, $y^{\left(t\right)}$ represents the real environment perception value at time step t, ${\hat{y}}^{\left(t\right)}$ represents the prediction result of the model at the corresponding time step, and T is the total number of time steps. ||·||^2^ denotes the L2 norm, which is used to measure the Euclidean distance between the predicted values and the true values, while the outer square term is used to calculate the squared prediction error. By minimizing this loss function, the model can gradually learn the mapping relationship between the human-light spatio-temporal interaction relationship and the environmental perception results.

During the model training process, the optimization method based on gradient descent is used to iteratively update the parameters. The goal can be expressed as:


$$\begin{array}{c} \Theta^{*}={argmin}_{\Theta }\;L \end{array}$$ (17)

where Θ represents the set of all learnable parameters in the model.

During the model training process, the network structure and training parameters were uniformly configured by comprehensively considering the model representation capability and computational complexity. The proposed GAT-STGNN model consists of two graph attention layers and one temporal attention fusion layer. The hidden feature dimension was set to 64, and the temporal modeling window length was set to 10. The hidden layers of the network adopt the Rectified Linear Unit (ReLU) activation function to enhance nonlinear feature representation capability. To reduce the risk of model overfitting, a Dropout layer was added after the feature fusion layer, with the dropout rate set to 0.2. Model training was performed using the Adam optimizer for parameter updating, with an initial learning rate of 0.001, a batch size of 64, and a maximum of 200 training epochs. During training, the validation loss was used as the monitoring criterion. When the validation loss did not decrease for 20 consecutive training epochs, early stopping was applied, and the model parameters with the best validation performance were saved. This strategy prevents the model from excessively fitting local temporal patterns during the training stage, enabling the model to focus more on stable spatio-temporal features in human-light interaction relationships rather than simply memorizing environmental variation patterns on specific dates. During the data preprocessing stage, the Min–Max normalization method was applied to the input features to map data with different scales into the range of [0,1], thereby improving the stability of model training.

### 3.4 Evaluation indicators

In order to comprehensively evaluate the modeling effect of the proposed human-light space-time graph neural network in indoor light environment perception tasks, this paper selects evaluation indicators from three aspects: prediction accuracy, error stability and overall fitting ability to quantitatively analyze the model performance.

Mean Squared Error (MSE) is used to measure the average squared deviation between model predictions and real observations. It is one of the most commonly used evaluation indicators in environment-aware continuous value prediction tasks. It is defined as:


$$\begin{array}{c} \text{MSE}=\frac{\text{1}}{T}\sum_{t=1}^{T}{\left({\hat{y}}^{\left(t\right)}-y^{\left(t\right)}\right)}^{\text{2}} \end{array}$$ (18)

Among them, y^(t)^ represents the real environment perception value at time step t, ${\hat{y}}^{\left(t\right)}$ represents the model prediction value, and T is the total number of time steps in the test set. The smaller the MSE value, the lower the overall prediction error of the model.

In order to enhance the interpretability of the error results at the physical dimension level, this article further introduces the root mean square error (RootMeanSquaredError, RMSE) as an auxiliary evaluation index, which is defined as:


$$\begin{array}{c} \text{RMSE}=\sqrt{\text{MSE}} \end{array}$$ (19)

Mean Absolute Error (MAE) is used to measure the average absolute magnitude of prediction errors. Compared with MSE and RMSE, it is less sensitive to abnormal error points. It is defined as:


$$\begin{array}{c} \text{MAE}=\frac{\text{1}}{T}\sum_{t=1}^{T}\left|{\hat{y}}^{\left(t\right)}-y^{\left(t\right)}\right| \end{array}$$ (20)

In order to evaluate the overall fitting ability of the model to the changing trend of environmental perception, this article introduces the coefficient of determination (Coefficient of Determination, R^2^) as an evaluation index, which is defined as:


$$\begin{array}{c} R^{\text{2}}=1-\frac{\sum_{t=1}^{T}{\left(y^{\left(t\right)}-{\hat{y}}^{\left(t\right)}\right)}^{\text{2}}}{\sum_{t=1}^{T}{\left(y^{\left(t\right)}-\overset{―}{y}\right)}^{\text{2}}} \end{array}$$ (21)

Among them, $\overset{―}{y}$ represents the mean value of the real environment perception value of the test set. The closer R^2^ is to 1, the stronger the model’s ability to explain changes in environmental perception.

## 4. Research results and analysis

### 4.1 Model performance evaluation

#### 4.1.1 Convergence analysis.

**Fig 3:**
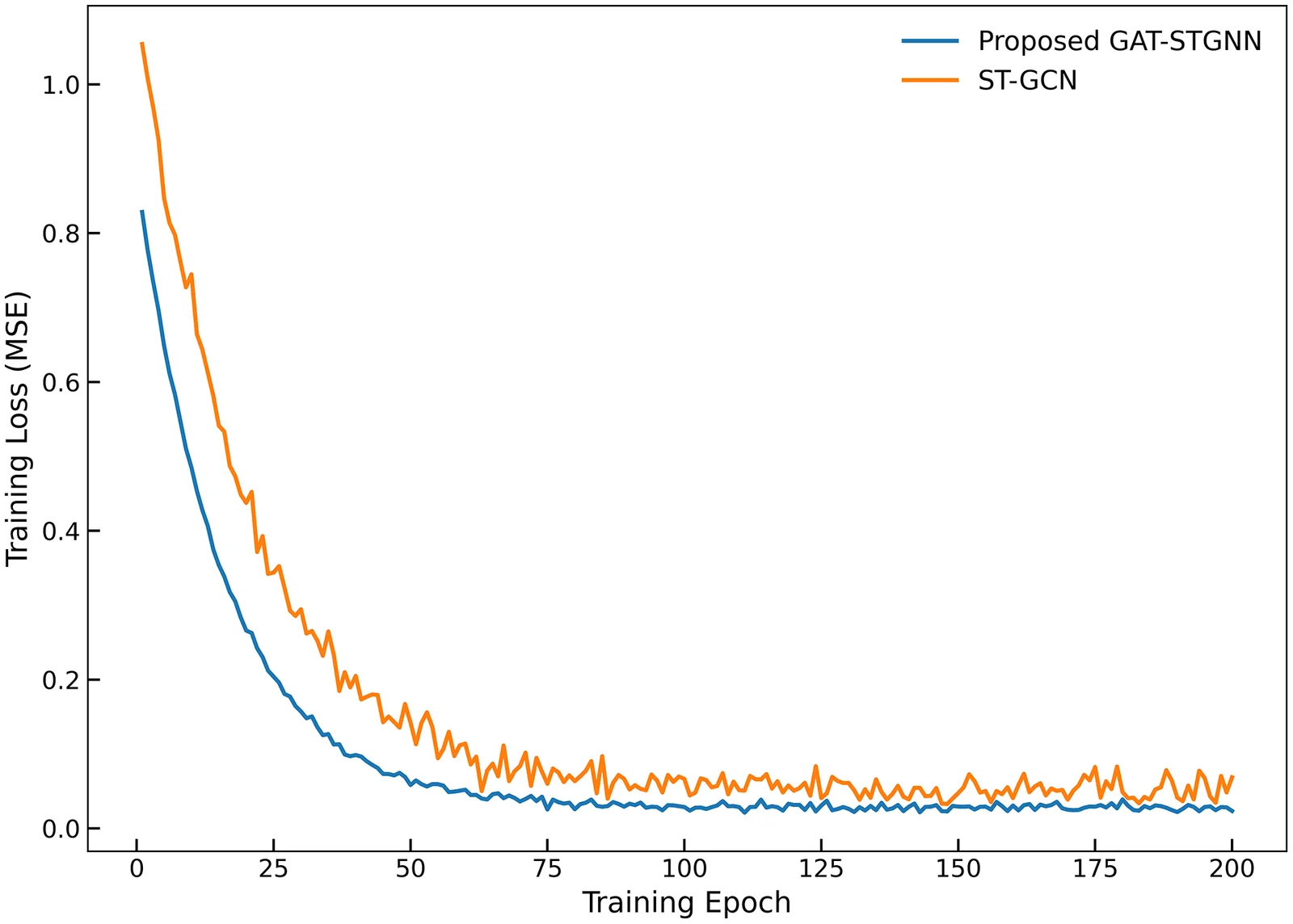
Comparison of convergence curves.

The results in Fig 3 show that both GAT-STGNN and Spatial-Temporal Graph Convolutional Network (ST-GCN) can achieve stable convergence within limited training rounds, indicating that the two models have good trainability under this task. In contrast, GAT-STGNN always maintains a lower training loss throughout the training process, and shows a faster loss decline trend in the early stages of training, indicating that it can effectively learn the human-light spatiotemporal relationship characteristics in the early stages of optimization. As the training progresses, the loss curve of GAT-STGNN gradually becomes stable, and the fluctuation amplitude in the middle and late stages is significantly smaller than that of ST-GCN, reflecting a more stable convergence behavior. Overall, the proposed model is superior to the comparative model in terms of convergence speed and optimization stability, providing a reliable basis for subsequent performance evaluation results.

#### 4.1.2 Overall performance analysis.

In order to comprehensively evaluate the modeling performance of GAT-STGNN in indoor light environment perception tasks, this article selects the following three models for comparison. ST-GCN, as a typical spatio-temporal graph convolution method, can depict the spatial structure and temporal evolution relationship between nodes; Attention-Based Spatial-Temporal Graph Convolutional Network (ASTGCN) introduces an adaptive spatio-temporal attention mechanism on this basis to enhance the modeling ability of key spatio-temporal dependencies; Graph Multi-Attention Network (GMAN) models complex spatio-temporal relationships through multiple graph attention structures, which represents the current more advanced spatio-temporal graph attention method. The above models form a progressive level from basic to advanced in terms of structural complexity and modeling capabilities, and can be used to systematically evaluate the performance advantages of this model. The following results are calculated based on multiple independent repeated experiments. All results presented below were obtained based on five independent repeated experiments.

To ensure fair comparisons among different models, the main structural parameters and training configurations of all models were uniformly set, with the specific parameters presented in Table 2. All models adopted the same data partition strategy, input features, and training schemes to ensure the fairness of performance comparisons. Among them, ST-GCN was mainly used to evaluate the modeling capability of traditional spatio-temporal graph convolution methods, ASTGCN and GMAN represented advanced methods incorporating spatio-temporal attention mechanisms and graph attention mechanisms, respectively, while GAT-STGNN further integrated human-light heterogeneous relationships and adaptive temporal modeling mechanisms.

**Table 2:** Configuration comparison of different models.

Model	Network architecture	Hidden dimension	Temporal modeling strategy	Attention mechanism	Optimizer	Learning rate
ST-GCN	Two-layer spatio-temporal graph convolutional layers	64	Temporal convolution	None	Adam	0.001
ASTGCN	Two-layer spatio-temporal graph convolutional layers with spatio-temporal attention modules	64	Temporal convolution with temporal attention	Spatial and temporal attention	Adam	0.001
GMAN	Multi-head graph attention layers with spatio-temporal attention modules	64	Temporal encoding module	Multi-head graph attention	Adam	0.001
GAT-STGNN	Two-layer heterogeneous graph attention layers with adaptive temporal attention layer	64	Temporal attention with a window length of 10	Human–light relationship-aware attention	Adam	0.001

**Fig 4:**
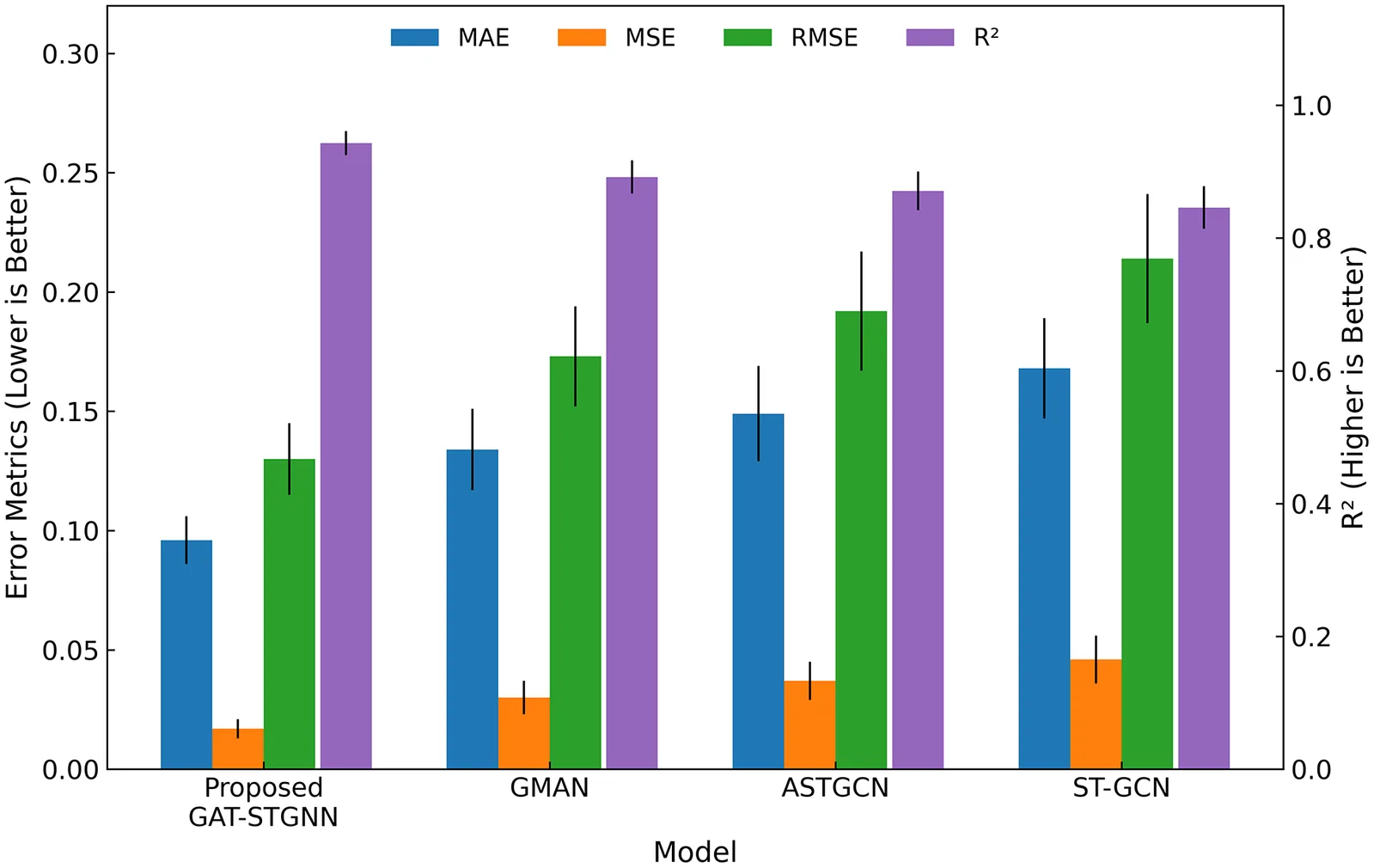
Overall performance comparison of different models. Note: MAE represents Mean Absolute Error, MSE represents Mean Squared Error, RMSE represents Root Mean Squared Error, and R^2^ represents the Coefficient of Determination. Lower values of MAE, MSE, and RMSE indicate better prediction performance, while higher values of R^2^ indicate better model fitting capability.

As shown in Fig 4, in terms of the mean absolute error (MAE) metric, GAT-STGNN achieved the lowest value of 0.096 ± 0.010, which was lower than those of GMAN (0.134 ± 0.017), ASTGCN (0.149 ± 0.020), and ST-GCN (0.168 ± 0.021). To further examine the statistical significance of the differences in model performance, a two-sided paired t-test was conducted on the results of five independent repeated experiments. All five experiments used the same data partitioning strategy, input features, and training configurations, with different random seeds assigned to each experiment. In each repeated experiment, the MAE values obtained by GAT-STGNN and each comparison model on the same test set constituted a pair of paired observations; therefore, each model comparison included five pairs of observations (n = 5). The statistical results showed that the differences in MAE between GAT-STGNN and GMAN, ASTGCN, and ST-GCN were statistically significant, with corresponding p-values of 0.018, 0.009, and 0.004, respectively. These results demonstrate that the performance improvements of the proposed model over the different comparison models are statistically reliable across repeated experiments. This result shows that in indoor light environment perception scenarios, this model can more accurately depict the mapping relationship between human body behavior changes and lamp status responses, effectively reducing the average prediction deviation introduced by the uncertainty of human-light interaction. At the same time, the standard deviation corresponding to its MAE is the smallest, indicating that the model’s perception results of the human-light relationship are highly stable under different time periods and data division conditions.

In terms of the mean square error (MSE) and root mean square error (RMSE) indicators, GAT-STGNN achieved the optimal results of 0.017 ± 0.004 and 0.130 ± 0.015 respectively, which were significantly better than GMAN’s 0.030 ± 0.007 and 0.173 ± 0.021, as well as the corresponding results of ASTGCN and ST-GCN. The significance test results indicated that the differences in the MSE and RMSE metrics between GAT-STGNN and GMAN, ASTGCN, and ST-GCN all reached statistically significant levels (p < 0.05). Since MSE and RMSE are more sensitive to large prediction deviations, this result shows that the model in this paper can effectively suppress large errors caused by individual abnormal human-light interaction states during the modeling process. This is particularly important for indoor light environment perception tasks, because sudden changes in human body position or lighting state switching often lead to instantaneous disturbances in environmental parameters, and GAT-STGNN can respond to such changes more robustly by jointly modeling the human-light spatial structure and temporal evolution characteristics.

In terms of the coefficient of determination (R²) metric, GAT-STGNN achieved the highest value of 0.943 ± 0.018, which was higher than those of GMAN (0.892 ± 0.025), ASTGCN (0.871 ± 0.029), and ST-GCN (0.846 ± 0.032). The statistical test results indicated that the differences in R^2^ between GAT-STGNN and each comparison model reached significant levels (p < 0.05). This result shows that the model in this paper can explain about 94.3% of the changes in environmental perception data, indicating that it can more fully capture the impact of human-light spatiotemporal interaction on the evolution of the light environment at the overall level. At the same time, the standard deviation corresponding to the R² index is small, which further reflects the consistency of the model’s fitting ability under different experimental conditions and provides reliable support for its application in actual indoor light environment design and control scenarios.

#### 4.1.3 Hyperparameter sensitivity analysis.

To further verify the rationality of the model parameter settings, a hyperparameter sensitivity analysis experiment was conducted in this study. Considering that the performance of the GAT-STGNN model is mainly affected by the utilization range of temporal information and feature representation capability, two key hyperparameters, namely the temporal window length and hidden feature dimension, were selected for analysis. During the experiments, the data partition strategy, optimizer, learning rate, and other training parameters were kept unchanged. Only the target hyperparameters were adjusted, and four metrics, including MAE, MSE, RMSE, and R², were used to evaluate the performance variations of the model.

The temporal window length determines the range of historical environmental states that can be utilized by the model. An excessively short temporal window may result in insufficient utilization of historical information, whereas an excessively long temporal window may introduce weakly correlated information and increase the learning burden of the model. Therefore, temporal window lengths of 5, 10, 15, and 20 were respectively configured for experiments, and the results are presented in Table 3.

**Table 3:** Performance variations of GAT-STGNN under different temporal window lengths.

Temporal window length	MAE	MSE	RMSE	R^2^
5	0.112 ± 0.013	0.023 ± 0.005	0.152 ± 0.017	0.921 ± 0.021
10	0.096 ± 0.010	0.017 ± 0.004	0.130 ± 0.015	0.943 ± 0.018
15	0.101 ± 0.012	0.019 ± 0.004	0.138 ± 0.016	0.936 ± 0.019
20	0.105 ± 0.013	0.021 ± 0.005	0.145 ± 0.017	0.931 ± 0.020

As shown in Table 3, with the increase in temporal window length from 5 to 10, the prediction performance of the model improved significantly, with MAE decreasing from 0.112 to 0.096 and R² increasing from 0.921 to 0.943. This indicates that appropriately increasing historical temporal information enables the model to more effectively learn the dynamic variation patterns during human-light interactions. When the temporal window length was further increased to 15 and 20, the model performance did not continue to improve but instead exhibited a certain degree of degradation. This suggests that excessively long temporal windows may introduce historical information with weak correlations to the current environmental state, thereby reducing the capability of effective feature extraction. Therefore, a temporal window length of 10 achieves a better balance between historical information utilization and model complexity.

The hidden feature dimension determines the representation capability of the model for human-light interaction relationships and environmental state features. To analyze the influence of different hidden dimensions on model performance, hidden feature dimensions of 32, 64, and 128 were respectively configured for experiments, and the results are presented in Table 4.

**Table 4:** Performance variations of GAT-STGNN under different hidden feature dimensions.

Hidden feature dimension	MAE	MSE	RMSE	R²
32	0.108 ± 0.012	0.021 ± 0.005	0.145 ± 0.016	0.931 ± 0.020
64	0.096 ± 0.010	0.017 ± 0.004	0.130 ± 0.015	0.943 ± 0.018
128	0.098 ± 0.011	0.018 ± 0.004	0.134 ± 0.015	0.940 ± 0.019

As shown in Table 4, when the hidden feature dimension increased from 32 to 64, all performance metrics of the model were improved, indicating that increasing the feature dimension can enhance the capability of the model to represent complex human-light spatio-temporal interaction relationships. However, when the hidden dimension was further increased to 128, the model performance did not continue to improve, and some metrics exhibited slight degradation. This may be attributed to the fact that an excessively high feature dimension increases the model parameter scale, making the model more likely to learn redundant features and consequently affecting its generalization capability. Therefore, considering both prediction accuracy and model complexity, this study finally selected a hidden feature dimension of 64 as the model configuration.

### 4.2 Spatiotemporal distribution characteristics of environmental perception results

**Fig 5:**
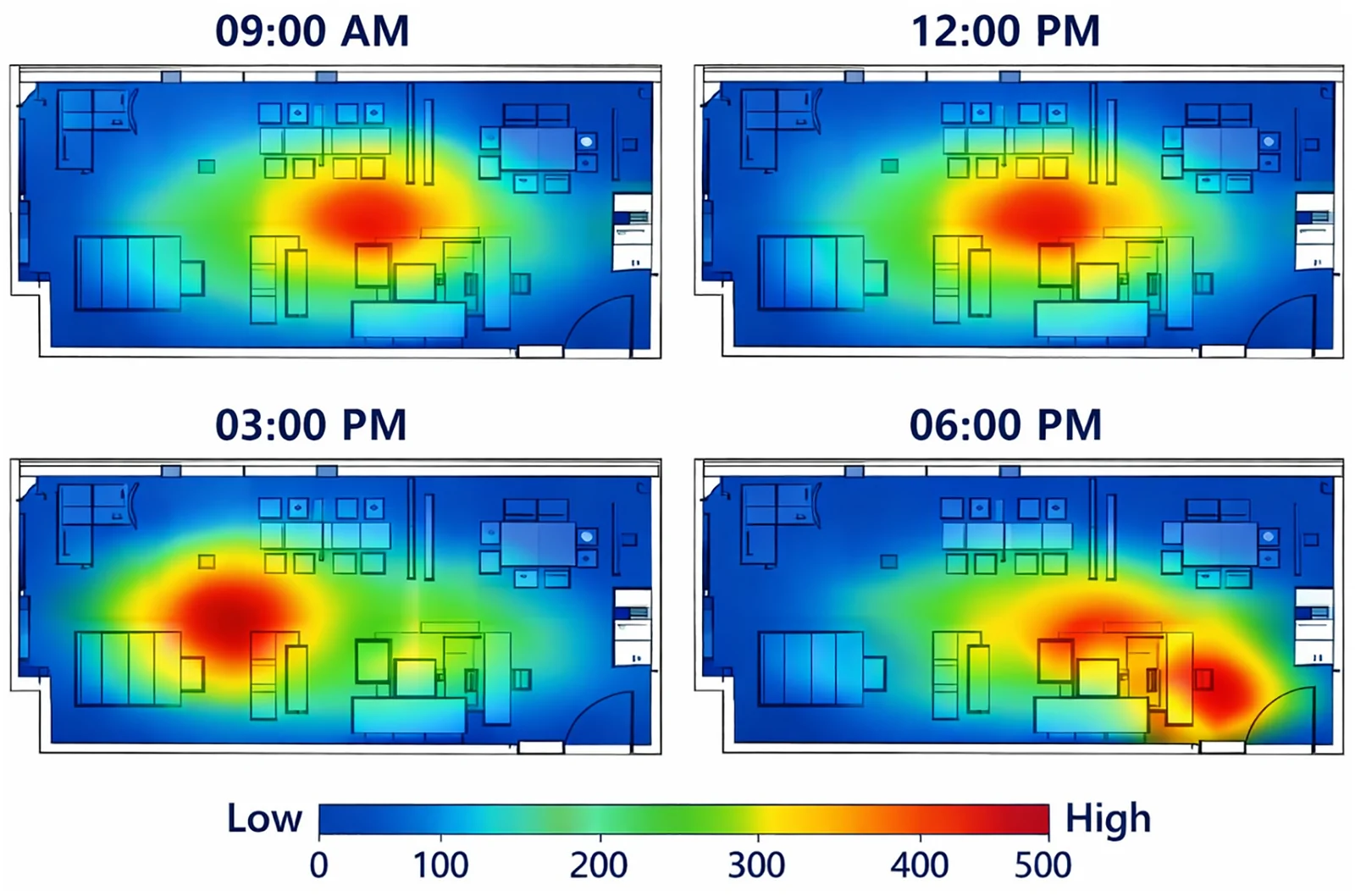
Spatiotemporal distribution diagram of environment perception results (unit: lx).

The data presented in Fig 5 have been inverse normalized and restored to the actual illuminance values (lx), which are used to intuitively reflect the differences between the model prediction results and the true environmental perception values. As shown in Fig 5, the environment perception results show significant non-uniform distribution characteristics in the indoor space and time dimensions. Overall, the predicted environmental perception intensity shows obvious concentration in space. High-value areas are mainly distributed in functional areas where people move frequently or stay for a long time, while the perception values corresponding to edge areas and low-activity areas are relatively low, indicating that the environmental perception results have a clear spatial structure. This distribution characteristic shows that the model can effectively distinguish the response differences in different spatial areas during human-light interaction, rather than generating uniform or random environmental perception output.

From the perspective of time evolution, the spatial thermal distribution at different moments shows a continuous and orderly changing trend. At 09:00AM and 12:00PM, the high-perception value areas are mainly concentrated in the indoor core activity area, and the spatial position remains relatively stable; by 03:00PM, the high-value areas have significantly expanded in spatial range, reflecting the increase in the scope of people’s activities and their enhanced response to the light environment; at 06:00PM, the high-perception value areas have further spatial migration and tend to be locally concentrated, reflecting the direct impact of changes in personnel distribution on environmental perception results. Between different time slices, the change process in the high perception value area is smooth and continuous, and no sudden changes occur, indicating that the model can stably depict the environmental evolution process driven by human-light interaction in the time dimension.

Combining the results of the space and time dimensions, it can be seen that the environmental perception distribution is not determined by a single lamp state or static spatial structure, but is formed under the joint action of the human-light space-time interaction relationship. The high perception value area dynamically adjusts with changes in people’s activity patterns, which verifies that the constructed human-light spatiotemporal relationship modeling can effectively characterize the human-driven response characteristics in the indoor light environment.

### 4.3 Generalization capability analysis under different indoor scenarios

To further validate the generalization capability of the GAT-STGNN model under different indoor environmental conditions, two additional test scenarios were introduced beyond the basic experimental scenario. These scenarios consider the effects of spatial scale variation and lighting organization changes on model performance. For Scenarios 2 and 3, data collection was conducted separately after the corresponding adjustments to the spatial structure and lighting configuration. The same sensing devices, sampling interval, data synchronization, and preprocessing procedures as those used in the baseline scenario were adopted to construct independent scenario-specific test datasets. Data from the two extended scenarios were not involved in the training or validation of the baseline model and were used exclusively as independent test data to evaluate the generalization capability of the model under changes in spatial structure and lighting configuration. All three scenarios adopted the same data acquisition method and model input format, with only the spatial structure or luminaire deployment conditions adjusted, to evaluate the adaptability of the model to different human-light interaction relationships.

Among them, Scenario 1 represents the basic office space, with dimensions of 8 m × 6 m × 3 m, containing 6 human nodes and 8 luminaire nodes. Scenario 2 represents an open-plan office space, where the spatial dimensions were adjusted to 10 m × 6 m × 3 m, the occupant activity area was expanded, and the spacing distribution of luminaires was modified. Scenario 3 represents a zone-based lighting office space, where luminaires were divided into multiple control zones to change the correspondence between occupant activity areas and luminaire responses. The experimental results are presented in Table 5.

**Table 5:** Generalization performance of GAT-STGNN under different indoor conditions.

Scenario	Description	MAE	MSE	RMSE	R^2^
Scenario 1	Basic office space (8 m × 6 m × 3 m)	0.096 ± 0.010	0.017 ± 0.004	0.130 ± 0.015	0.943 ± 0.018
Scenario 2	Open-plan office space (10 m × 6 m × 3 m, enlarged activity area)	0.108 ± 0.012	0.021 ± 0.005	0.145 ± 0.017	0.928 ± 0.020
Scenario 3	Zoned lighting office space (multiple lighting control areas)	0.103 ± 0.011	0.019 ± 0.004	0.138 ± 0.016	0.935 ± 0.019

Overall, GAT-STGNN maintained relatively stable prediction performance in both extended scenarios based on newly collected independent data. Since the data for Scenarios 2 and 3 were collected independently from the baseline scenario, with actual changes in spatial scale, occupant activity range, or luminaire organization, these results reflect the cross-scenario adaptability of the model when applied to new indoor environment configurations. This further indicates that the human–light spatio-temporal relationships learned by the model exhibit a certain degree of transferability across environments, rather than being effective only for the specific spatial layout and lighting configuration of the baseline scenario. It should be noted that the current independent validation is still limited to office spaces and two extended configurations. Therefore, the results support the generalization capability of the model within the tested indoor environments, but do not imply equivalent generalization performance across all building spaces and lighting conditions.

As shown in Table 5, GAT-STGNN maintained stable prediction performance under different indoor conditions. Compared with the basic scenario, Scenario 2 involved changes in the spatial relationships between human nodes and luminaire nodes due to the expansion of spatial scale. The MAE increased from 0.096 ± 0.010 to 0.108 ± 0.012, while R² decreased from 0.943 ± 0.018 to 0.928 ± 0.020. These results indicate that spatial structure variations have a certain impact on model prediction performance. However, the overall performance degradation was limited, demonstrating that the model can learn transferable spatial correlation features.

After adopting zone-based lighting in Scenario 3, the organizational relationships among luminaire nodes changed. Nevertheless, the model still achieved an MAE of 0.103 ± 0.011 and an R² of 0.935 ± 0.019, which were close to the results of the basic scenario. This indicates that the heterogeneous graph attention mechanism can adaptively adjust feature weights according to the dynamic relationships between humans and luminaires, thereby adapting to different lighting configuration conditions.

Overall, GAT-STGNN exhibited strong prediction stability under different spatial conditions and lighting organization modes, validating the generalization capability of the model for different indoor human-light interaction patterns.

## 5. Discussion

### 5.1 Interpretability analysis of attention mechanisms

**Fig 6:**
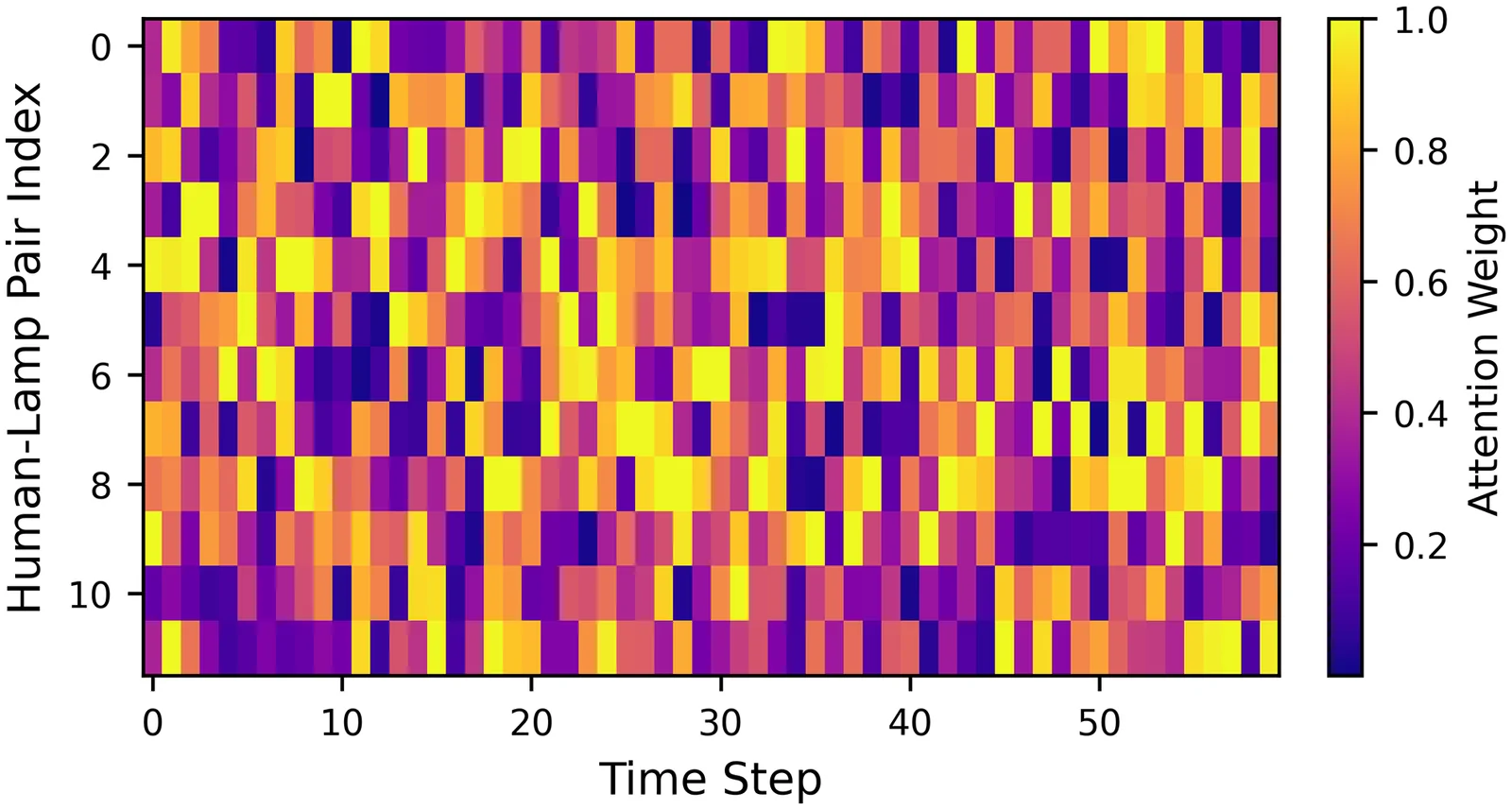
Distribution of human-light spatio-temporal attention weights.

To further analyze the learning mechanism of the proposed GAT-STGNN model for human-light interaction relationships, this study conducted an interpretability analysis of the spatio-temporal attention weights obtained during model training. As shown in Fig 6, the human-light spatio-temporal attention weights exhibit distinct distribution characteristics across both temporal and relational dimensions, indicating that the model does not treat the connection relationships between all human nodes and luminaire nodes equally. Instead, it can adaptively adjust the importance of different interaction relationships according to their influence on environmental perception results.

From the temporal dimension, the attention weights exhibit continuous variation characteristics, indicating that the model can dynamically adjust the contribution of different time steps to the current environmental perception results based on historical state information. Some highly correlated human-light node pairs maintain high attention weights within continuous temporal windows, suggesting that when stable associations are formed between human positions and luminaire responses, the model can continuously focus on such interaction information. Meanwhile, during periods of occupant movement or luminaire state changes, the attention weights of some node pairs increase significantly, indicating that the model can capture critical interaction processes caused by behavioral changes or lighting adjustments. This result demonstrates that the adaptive temporal attention mechanism can effectively identify the differences in the influence of various historical states on current lighting environment variations, rather than simply relying on fixed temporal windows for feature extraction.

From the spatial relationship perspective, significant differences exist in the attention weights between different human nodes and luminaire nodes, indicating that the heterogeneous spatial attention mechanism can learn human-light relationships with practical physical significance. For luminaire nodes that are closer to human activity areas and have stronger correlations with state changes, the model assigns higher attention weights, demonstrating that interactions between these nodes play a more important role in environmental perception results. In contrast, for node relationships with weaker spatial correlations or limited contributions to the current environmental state, the model can reduce their weight influence, thereby minimizing interference from redundant information. This relationship-importance-based feature selection approach enhances the capability of the model to represent key interaction structures in complex indoor lighting environments.

Furthermore, the differentiated distribution of attention weights not only improves the interpretability of the model prediction process but also provides insights into the mechanisms underlying indoor lighting environment variations. Compared with traditional spatio-temporal models that only output prediction results, the proposed model can identify key interaction relationships that significantly influence lighting environment changes through the attention weights between human and luminaire nodes, thereby revealing the dynamic coupling patterns between human behaviors and lighting responses. From the perspective of intelligent lighting design, attention weight analysis can assist in identifying key influencing factors in different spatial regions and temporal periods, providing references for lighting zoning optimization, luminaire state adjustment, and personalized lighting strategy development. In practical smart building applications, this interpretability capability can support lighting systems in dynamically adjusting according to occupant activities and spatial usage conditions, reducing ineffective lighting adjustments while improving environmental comfort, and providing interpretable data support for human-oriented intelligent lighting environment design.

### 5.2 Comparative analysis with existing studies

This study uses the Graph Attention Spatiotemporal Graph Neural Network (GAT-STGNN) to model the human-light interaction relationship in the indoor light environment. It uniformly represents human behavior and lamp response as a heterogeneous spatiotemporal graph, and jointly learns its evolution characteristics in the space and time dimensions. By introducing relationship-aware spatial attention and adaptive temporal attention mechanisms, the model can highlight key human-light interactions that contribute more to light environment changes. Experimental results show that this method has high accuracy and stability in environmental perception prediction, and can effectively deal with the uncertainty caused by changes in personnel behavior and lamp status. At the same time, it reveals the main spatiotemporal characteristics of indoor light environment response driven by human factors.

From the comparison with existing research, it can be seen that this paper not only inherits the basic method of indoor environment spatio-temporal diagram modeling in terms of modeling of human-light spatio-temporal relationship, but also forms targeted innovations at the level of modeling objects and mechanisms. Zhang et al. [23] abstracted indoor space and multi-source environmental data into a graph structure and verified the effectiveness of joint modeling of spatial topology and time evolution in improving the accuracy and stability of environmental parameter prediction, providing a methodological basis for this article. However, its research mainly focuses on the spatiotemporal distribution of environmental parameters themselves, and does not include human behavior as a structural element in the model. On this basis, this paper further introduces human body nodes and lamp nodes into the graph structure at the same time, making human-light interaction the core modeling object that drives changes in the light environment, thereby expanding the existing homogeneous environment node modeling framework. The interpretable spatiotemporal graph neural network proposed by Tang et al. [19] emphasizes the role of attention weights in revealing key spatial relationships and temporal dependencies, and its conclusion is consistent with the difference in the importance of human-light interactions observed in the attention weight analysis in this paper. The difference is that this article does not enhance the model interpretability after the fact, but explicitly introduces the human-light relationship type encoding in the spatial attention calculation stage, so that the attention weight directly corresponds to the specific interaction semantics, and achieves a more fine-grained depiction of the human-light relationship. Huang et al. [8] proved the key role of human factors in environmental prediction by integrating occupational information and environmental variables in large-scale indoor spaces. This conclusion is consistent in direction with the research judgment of this article. However, compared to its general modeling idea for multi-variable joint prediction, this paper focuses more on light environment design scenarios. Focusing on the interactive relationship of “human-light fixtures” with clear physical meaning, it introduces adaptive temporal attention to characterize the time imbalance characteristics of light environment response, and explains the robust performance of the model under behavioral mutation or lighting switching scenarios from the mechanism level. To further compare the performance differences between the proposed method and existing spatio-temporal prediction methods based on graph neural networks for indoor environments, this study conducted a comparative analysis between these studies and the proposed method. The results are presented in Table 6.

**Table 6:** Performance comparison between the proposed method and related studies.

Study	Reported results
Zhang et al. [23]	The RMSE of Graph Attention Network–Recurrent Neural Network (GAT-RNN) was 0.39 ± 0.14 °C. After applying the prediction-based control strategy, the proportion of time within Level I thermal comfort increased from 36.5% to 81.3%, and the proportion within Level I and II thermal comfort increased from 94.2% to 96.4%.
Tang et al. [19]	On the PeMS4 dataset: MAE = 18.57, RMSE = 30.14, Mean Absolute Percentage Error (MAPE)=12.13%; on the PeMS7 dataset: MAE = 20.00, RMSE = 33.45, MAPE = 8.51%; on the PeMS8 dataset: MAE = 14.59, RMSE = 23.91, MAPE = 9.80%. For crime prediction, the MAE and MAPE were 0.9095 and 0.5154 on New York City (NYC) dataset, and 1.0307 and 0.5016 on Chicago (CHI) dataset, respectively.
Huang et al. [8]	Attention-Based Spatial-Temporal Graph Convolutional Gated Recurrent Unit (AST-GCGRU) improved the overall prediction accuracy by 28.5% compared with baseline models. The prediction accuracy improvements for CO_2_ and occupant nodes were 14.9% and 27.1%, respectively. The robustness performance was improved by 11.3% compared with Graph Convolutional Gated Recurrent Unit (GCGRU).
This study (GAT-STGNN)	The proposed GAT-STGNN achieved MAE = 0.096 ± 0.010, MSE = 0.017 ± 0.004, RMSE = 0.130 ± 0.015, and R² = 0.943 ± 0.018.

As shown in Table 6, existing studies have demonstrated the effectiveness of graph neural networks in dynamic indoor environment prediction tasks. However, most of these studies mainly focus on single environmental variables, such as temperature, CO_2_ concentration, or occupant number, while insufficient attention has been given to the heterogeneous interaction relationships between human behaviors and lighting systems. In contrast, the proposed GAT-STGNN further represents human nodes and luminaire nodes as a unified heterogeneous spatio-temporal graph and learns human-light interaction processes through relationship-aware spatial attention and adaptive temporal attention mechanisms. Therefore, it is more suitable for human-oriented indoor lighting environment perception tasks.

The main contribution of this paper is to provide a generalizable system modeling method for human-centered indoor environment research. Compared with traditional modeling ideas centered on environmental parameters or equipment status, this paper highlights the structural role of human-light interaction in the formation of light environment. It not only improves the accuracy and stability of environmental perception prediction, but also provides an interpretable modeling tool for revealing the dynamic response mechanism of light environment driven by human factors, and provides a new technical path for related research on intelligent lighting and human-centered design.

Although this paper verifies the effectiveness of the proposed method based on real indoor scene data, there are still certain limitations. First of all, the model input mainly relies on basic information such as human body position and lamp operating status, and has not yet introduced richer human attributes or subjective perception variables, which limits the ability to depict individual differences and high-level light environment experiences to a certain extent. Secondly, the model mainly focuses on offline training and prediction, and its applicability in real-time control or online adaptive adjustment scenarios still needs to be further verified. Finally, the current study does not involve nighttime lighting scenarios under completely absence of natural light conditions. Since occupant activity patterns, lighting control strategies, and visual perception requirements may differ in nighttime environments, the applicability of the model in nighttime scenarios still requires further validation.

## 6. Conclusion

Aiming at the problem that it is difficult to simultaneously depict the spatial structure and temporal evolution of the human-light interaction relationship in indoor light environment design, this paper proposes a human-light spatiotemporal relationship modeling and environment perception method based on graph attention spatiotemporal graph neural network, and verified it. The main conclusions are as follows.

First, the proposed GAT-STGNN model demonstrates high environmental perception prediction capability on the real indoor scene dataset. The MAE, MSE, and RMSE values reached 0.096 ± 0.010, 0.017 ± 0.004, and 0.130 ± 0.015, respectively, while R² reached 0.943 ± 0.018. The overall performance was superior to that of comparison models such as ST-GCN, ASTGCN, and GMAN. The results indicate that incorporating human-light heterogeneous relationship modeling and dynamic temporal feature learning can effectively improve the capability of the model to represent complex indoor lighting environment variation processes, while enhancing the stability and reliability of environmental perception results.

Second, the environmental perception results show continuous and reasonable distribution characteristics in the spatial and temporal dimensions. The prediction results show that areas with high perceived intensity are mainly concentrated in areas with frequent human activities, and migrate smoothly in space over time. The overall evolution process is consistent with the human activity pattern, which verifies that the constructed human-light spatiotemporal relationship model can stably depict the dynamic response process of the light environment driven by human factors.

From the perspective of practical applications, the proposed method can dynamically perceive indoor lighting environment states based on the relationship between occupant position variations and luminaire state responses, providing real-time state information support for subsequent adaptive lighting control. In smart building scenarios, this method can assist lighting systems in dynamically adjusting according to user activity requirements, reducing unnecessary lighting energy consumption while ensuring visual comfort, and providing technical support for personalized intelligent lighting environment design.

Follow-up research can be expanded in multiple directions. First, multi-modal human factors data such as physiological perception, behavioral preferences or subjective comfort evaluation can be integrated to further enrich the semantic level of human-light interaction modeling, thereby improving the depth of the model’s depiction of light environment experience. Second, we can explore embedding this model into intelligent lighting systems to implement an online learning mechanism for real-time perception and dynamic control, so as to promote the application of research results in actual indoor light environment design and operation management. Third, the data acquisition period can be further extended by incorporating indoor lighting operation data under nighttime conditions without natural light. The effects of variations in nighttime occupant activity patterns, lighting control strategy adjustments, and differences in visual perception requirements on human-light interaction relationships can be comprehensively considered, thereby improving the generalization capability of the model across different temporal scenarios.

## Supporting information

S1 Data Minimal dataset. The dataset contains the experimental data supporting the analyses and findings of this study.
(RAR)
